# In-situ abiogenic methane synthesis from diamond and graphite under geologically relevant conditions

**DOI:** 10.1038/s41467-021-26664-3

**Published:** 2021-11-04

**Authors:** Miriam Peña-Alvarez, Alberto Vitale Brovarone, Mary-Ellen Donnelly, Mengnan Wang, Philip Dalladay-Simpson, Ross Howie, Eugene Gregoryanz

**Affiliations:** 1 Centre for Science at Extreme Conditions and School of Physics and Astronomy, University of Edinburgh, Edinburgh, UK; 2 Dipartimento di Scienze Biologiche, Geologiche e Ambientali (BiGeA), Alma Mater Studiorum Università di Bologna, Piazza di Porta San Donato 1, 40126 Bologna, Italy; 3 Sorbonne Université, Muséum National d’Histoire Naturelle, UMR CNRS 7590, IRD, Institut de Mináralogie, de Physique des Matáriaux et de Cosmochimie, IMPMC, 75005 Paris, France; 4 Center for High Pressure Science and Technology Advanced Research (HPSTAR), Shanghai, China; 5 Key Laboratory of Materials Physics, Institute of Solid State Physics, Chinese Academy of Sciences, Hefei, China

**Keywords:** Geochemistry, Mineralogy

## Abstract

Diamond and graphite are fundamental sources of carbon in the upper mantle, and their reactivity with H_2_-rich fluids present at these depths may represent the key to unravelling deep abiotic hydrocarbon formation. We demonstrate an unexpected high reactivity between carbons’ most common allotropes, diamond and graphite, with hydrogen at conditions comparable with those in the Earth’s upper mantle along subduction zone thermal gradients. Between 0.5-3 GPa and at temperatures as low as 300 °C, carbon reacts readily with H_2_ yielding methane (CH_4_), whilst at higher temperatures (500 °C and above), additional light hydrocarbons such as ethane (C_2_H_6_) emerge. These results suggest that the interaction between deep H_2_-rich fluids and reduced carbon minerals may be an efficient mechanism for producing abiotic hydrocarbons at the upper mantle.

## Abstract (web summary)

Using diamond anvil cell and high temperature experiments, this work proves that the interaction between deep hydrogen rich fluids and reduced carbon minerals may be an efficient mechanism for producing abiotic hydrocarbons at the upper mantle’s pressures and temperatures.

## Introduction

The process of abiotic hydrocarbon formation in the deep Earth is still contested, despite being central in geo-biological processes and potential natural energy sources^1,2^. Light hydrocarbons of abiotic origin have been identified in an increasing number of geological fluids in the Earth’s lithosphere^3–6^. Methane has also been detected within deep diamonds, suggesting the presence of abiotic hydrocarbons at mantle depths^7–9^. However, their formation mechanisms and distribution, as well as their possibility to degas towards the crust and the atmosphere, remain largely unconstrained. The abiotic formation of stable light hydrocarbons, such as methane (CH_4_), was mainly considered to occur through reduction paths and, generally, in the presence of oxygen carrying species such as carbon monoxide (CO) or carbon dioxide (CO_2_) through the so-called Fischer–Tropsch Type reactions^10–14^.

**Fig. 1:**
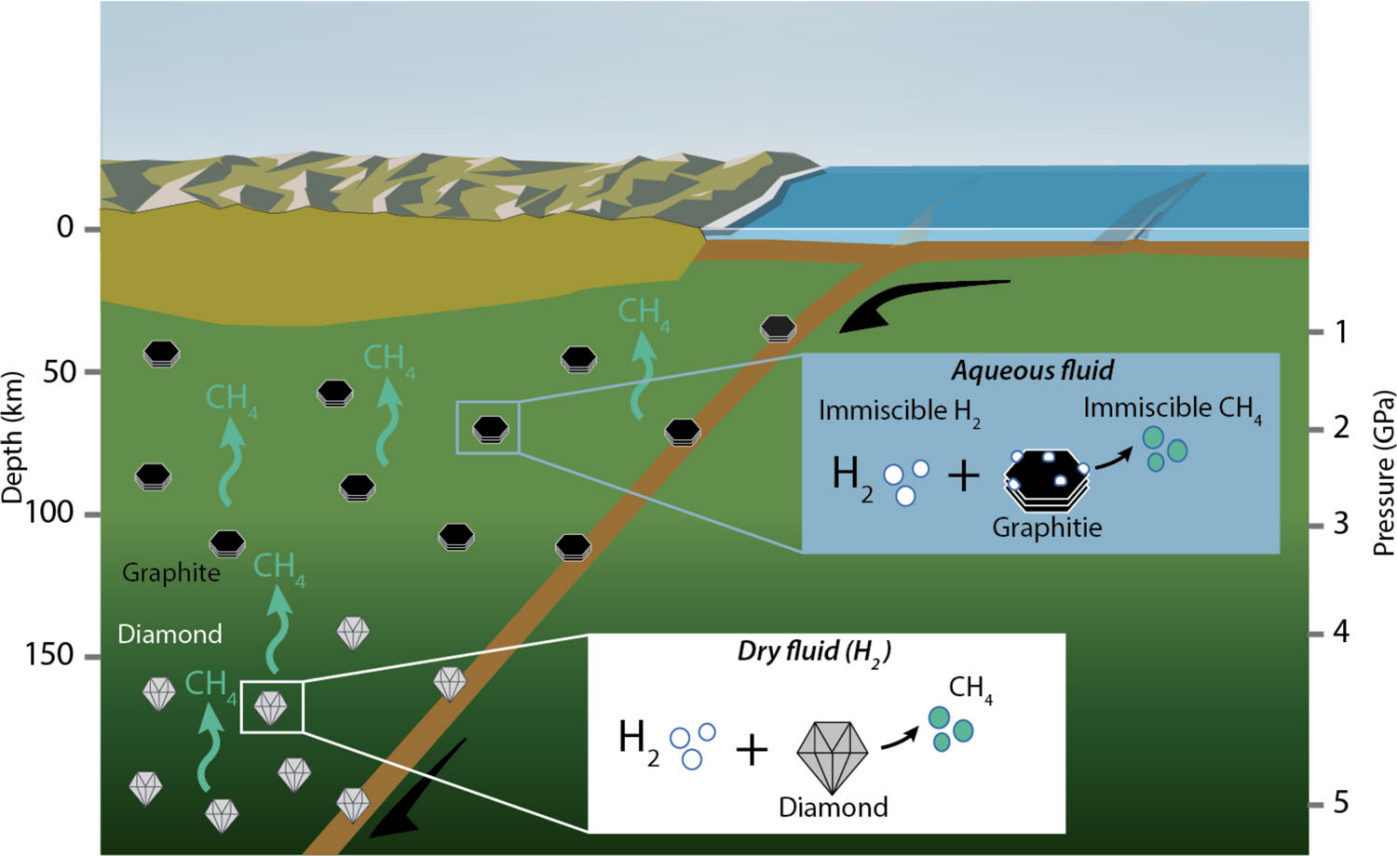
Sketch modified after Fig. 1 in Li et al.^52^ summarizing the implications of this study on the genesis of abiotic CH_4_ in the upper mantle. Below about 140 km depth (4 GPa), the immiscibility of H_2_ in aqueous fluids^22^ can promote interactions between H_2_ gas and graphitic carbon, leading to the formation of CH_4_ (Fig. 3). This condition is plausible in reducing settings with low oxygen concentrations within the upper mantle^17^ or where reducing conditions are generated in subduction zones^25^. At greater depths in the diamond stability field, the oxygen fugacity is predicted to be lower^14^ and dry H_2_ fluids are more common^7, 8^ and interact with diamond to form abiotic CH_4_ (Fig. 1).

In the Earth’s interior, diamond and graphite are the major carbon reservoirs^14^, whereas hydrogen (H_2_) is among the most volatile fluid elements. Graphite and other forms of carbonaceous materials are dominant at depths between 50 and 140 km (2–4 GPa)^14–16^, whereas deeper than 140 km depth (4 GPa) diamond becomes stable^17^. In Fig. 1, we summarize the relationship between pressure, in deep in the Earth’s mantle, and the evolution of the distribution of graphite and diamond, together with hydrogen and methane clusters.

Methane may be a fundamental component of upper mantle fluids^14^. It reacts under high pressure, forming long-chain hydrocarbons, and then it is predicted to eventually dissociate into diamond, graphitic carbon and hydrogen^18–21^. Methane at depths could co-exist with molecular hydrogen (H_2_) and small amounts of light hydrocarbons and different carbon allotropes^10,13,14^. However, the origin of methane in the upper mantle remains largely unconstrained^2^.

Reactions between H_2_-rich fluids and carbon-bearing parent minerals may be effective to produce methane and other hydrocarbons abiotically. At upper mantle conditions, H_2_ may be present and immiscible in aqueous fluids, and react with condensed carbon minerals^22–26^. Water–rock interactions at subduction zone conditions may also be effective in generating H_2_-rich fluids^6,27–31^, also in the presence of graphite^25,32,33^. Molecular hydrogen may also be present in minerals at upper mantle conditions^34^. Recent analysis of fluid inclusions in super-deep diamonds indicates that H_2_ may represent a significant component of upper mantle fluids in the presence of diamond^7,8^. Yet, reactivity between diamond and H_2_ at upper mantle conditions has not been contemplated as a source of abiogenic hydrocarbons.

Here we investigate abiotic methane production from the precursors of pure H_2_ and condensed carbon minerals such as diamond and graphite. We conduct in situ experiments using a resistively heated diamond anvil cell (DAC) at pressure and temperature conditions in the range of 0.5–5 GPa and 300–730 °C, and use Raman spectroscopy as the diagnostic tool. Most of the investigated conditions are consistent with Earth’s upper mantle and subduction zone *P*–*T* (pressure and temperature) gradients^35^. We find that at these mild *P*–*T* conditions, diamond and graphite react readily with H_2_ to form methane and other light hydrocarbons, such as ethane (C_2_H_6_). This demonstrates that the reaction between condensed carbon phases and H_2_ could be an important source of abiotic hydrocarbons, which should be considered in the deep Earth’s carbon cycle.

## Results

### Diamond and hydrogen

**Fig. 2:**
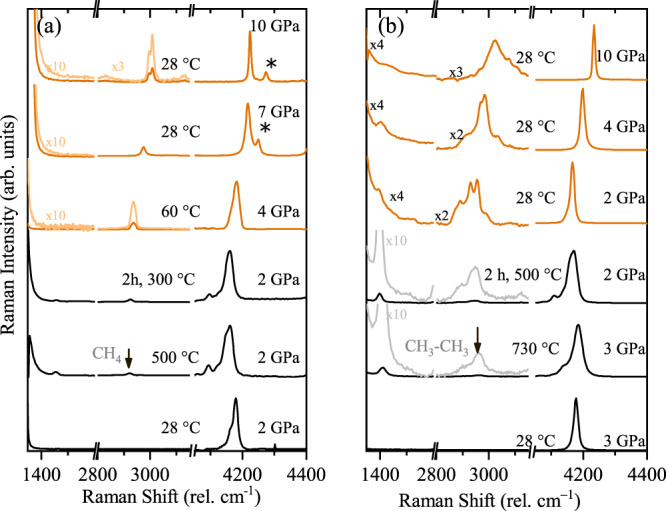
Raman spectra of resistive heating of hydrogen in a diamond anvil cell at selected pressures and temperatures. **a** Up to a maximum temperature of 500 °C and held at 300 °C for 2 h and after cooling down. Orange spectra correspond to the cooled down sample. **b** Up to a maximum temperature of 730 °C and held at 500 °C for 2  h and after cooling down. Orange spectra correspond to cooled down sample. The CH stretching modes of CH_4_ (**a**) and C_2_H_6_ (**b**) appear between 2900 and 3000 cm^−1^. In the cooled down sample of experiment **b**, the CH wagging mode is seen at around 1480 cm^−1^. The band at around 4200 cm^−1^ corresponds to the vibrational mode of H_2_ (*Q*_1_^50^, this is accompanied by the rotational + vibrational band (*Q*_1_ + *S*_0_) visible in liquid hydrogen. At high temperatures, there is another band at lower frequency, which corresponds to the thermally populated second vibrational state of hydrogen^51^. Asterisks mark the vibrational mode from the CH_4_-H_2_ van der Waals compounds^41^.

At room temperature and at pressures between 2 and 3 GPa, Raman measurements show only the characteristic spectrum of the H_2_ sample, and that of the diamond anvils (Fig. 2). Heating hydrogen in a DAC at 2 GPa (which corresponds to Earth depths of about 70 km^36^, see Fig. 1) to temperatures of 500 °C, we observe a new Raman band appearing at ~2900 cm^−1^ within ~20 min (see Fig. 2). This new band can be detected uniformly across the sample chamber. Repeating measurements at 3 GPa (below or around 70 km depth, see Fig. 1) and holding the sample at lower temperatures of 300 °C for a period of 2 h, the same results are observed; a new band at 2900 cm^−1^ appears and its intensity grows with time. This new mode coincides with the most intense C-H vibrational stretching mode of methane, indicating abiotic methane production from the only elements present in the experimental chamber: hydrogen and diamond.

High-temperature studies of dense methane have yielded other light hydrocarbons such as ethane (C_2_H_6_), propane (C_3_H_8_), butane (C_4_H_10_) and isobutane (C_4_H_10_)^19^. In our experiments, increasing temperature to 730 °C at 3 GPa leads to the growth of more complex vibrational excitations, centred around ~2950 cm^−1^ (Fig. 2b). The intensity of these modes increases with time if the sample is held at above 500 °C for 2 h. By comparing the obtained spectrum with spectra reported for hydrocarbons in the literature^37–39^, we can identify the additional product as ethane (C_2_H_6_). Upon temperature quenching, samples were subsequently compressed up to 30 GPa at room temperature. The evolution of the vibrational spectra and their frequencies vs. pressure are in good agreement with those of methane and ethane (Supplementary Fig. 2)^37,40,41^. We also note that at above 5 GPa, we observe an additional vibrational mode, which is present neither in pure methane nor in hydrogen (see mode indicated by asterisks in Fig. 2a). This new mode has previously been interpreted as being a feature of a CH_4_-H_2_ van der Waals compounds^41^. Experiments were repeated using deuterium as a precursor instead of hydrogen, in which we observed the formation of CD_4_ (Supplementary Fig. 1). The presence of CD_4_ after heating is evidence that the reaction is between the D_2_ sample and diamond, and not from residuals and/or a contaminant from the preparation process.

**Fig. 3:**
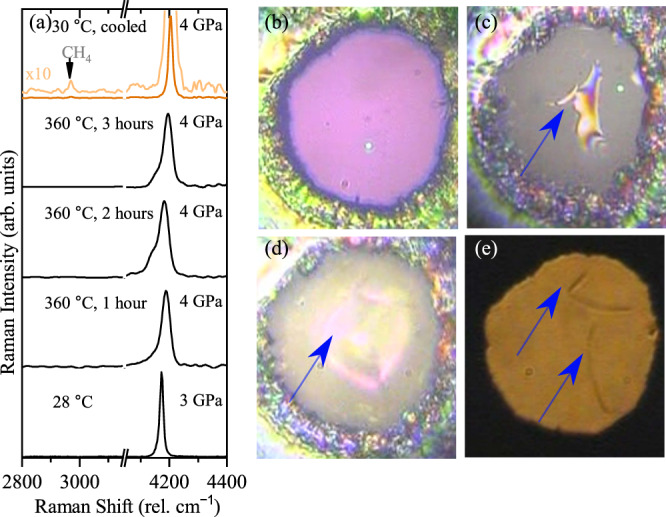
Hydrogen sample resistively heated during heating and cooling process at 3 GPa, using a diamond anvil cell whose culets and gasket hole had been coated with 300 nm Al_2_O_3_ with chemical vapour deposition. **a** Representative Raman spectra during heating and cooling (orange spectrum). **b** Image taken in transmitted and reflected light, while being heated at 360 °C for 1 h. **c** Image of the sample at 360 °C for 3 h, the image has been taken in reflected light so the region of the chamber where coating is becoming damaged is seen. **d** Image of the sample after cooling in transmitted light. **e** Image of the sample after cooling down only in transmitted light. Blue arrows are used to point the regions of the damaged Al_2_O_3_ layer; green spots are due to the laser beam.

We performed three control experiments to eliminate the possibility of carbon contaminants in the sample chamber, whereby the gasket and diamonds were insulated from the hydrogen sample with aluminium oxide (Al_2_O_3_) (see Fig. 3). Al_2_O_3_ has been shown to provide a protective layer that slows down hydrogen diffusion into diamond at high pressures and temperatures^42^. Therefore, it could preclude the formation of CH_4_ from the diamond anvil and hydrogen. Inspection of the optical images of the sample chamber after 1 h at 360 °C and 4 GPa reveals that the coating was still pristine (Fig. 3b) and no methane was observed spectroscopically. However, after 3 h at this temperature, part of the coating began breaking up and detaching from the diamond (Fig. 3c), becoming more visible during the cooling of the sample (Fig. 3d–e). This deterioration of the coating with temperature and time enabled hydrogen to reach the diamonds, forming methane on contact. We also considered that the transition metals from which the gaskets are made, could catalyse the reactions^43^. We conducted several heating runs with different gasket materials such as rhenium (Re) and tungsten (W), and gasket liners, e.g., gold (Au) and Al_2_O_3_ (see Supplementary Table 1 for a list of the materials used). We observed that, regardless of the gasket and gasket insert materials, if the diamonds are not protected by Al_2_O_3_, CH_4_ and/or C_2_H_6_ are always produced.

### Graphite, glass-like carbon and hydrogen

**Fig. 4:**
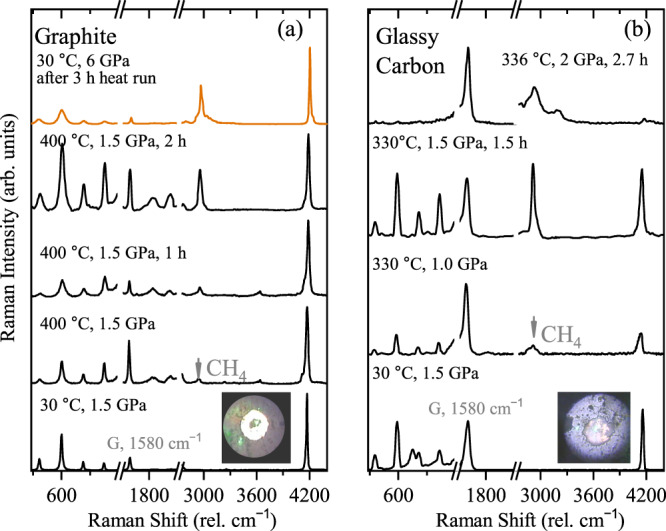
Resistive heating of hydrogen in a diamond anvil cell. **a** Graphite loaded together with H_2_, spectra at selected temperatures and pressure. Spectra have been normalized to the H_2_ stretching mode, around 4200 cm^−1^. Orange spectrum correspond to the quenched sample. **b** Glassy-like carbon loaded together with H_2_, spectra are normalized to the G band characteristic of carbonaceous materials at around 1580 cm^−1^. Inserted images correspond to the sample within the diamond anvil cell chamber during the experiment.

As graphite may be an important component of subducted sedimentary rocks^44–46^, we repeated our experiments by adding graphite into the sample chamber. These experimental runs yielded identical results producing larger amounts of methane on a shorter timescale than with the diamond precursor. We also explored the reactivity of disordered carbonaceous materials using a glass-like form of carbon (for which thermodynamic data are available^46^), which may be common below 500–600 °C in subduction zones^15^. Figure 4 shows the Raman spectra between 1.0 and 1.5 GPa during heating cycles of (a) H_2_-graphite and (b) H_2_-glassy-like carbon. In both cases, there is a rapid growth of the C-H stretching mode of methane with time. Similar to the methane production from diamond, CH_4_ forms compounds with H_2_ on compression of the quenched sample^41^.

## Discussion

In each of the graphite and glassy carbon experiments, similar amounts of samples were used. Comparing the intensities of methane produced by different carbonaceous samples, one can conclude that glassy-like carbon and graphite are naturally more reactive to H_2_ than diamond (see Supplementary Fig. 4 to compare the relative intensities of methane peaks generated from the different starting materials). Of course, this statement is tentative and approximate, as the graphite and glassy-like carbon measurements also contain a contribution from the methane formed from the diamond anvils and there are other factors such as background and hydrogen accessibility to the carbonaceous sample that influence the experiment. Nevertheless, this suggests that graphitic carbon materials can act as an efficient reactant for abiotic CH_4_ formation in upper mantle and crustal environments.

Condensed carbon reservoirs and their mobilization in deep fluids may represent a key to unravelling deep carbon recycling. We have shown that under dry conditions, and in the absence of oxygen or a catalyst, methane is formed from diamond, graphite or glassy carbon and hydrogen, at conditions comparable to the outer layers of the Earth’s mantle (depths of 18–160 km, pressures of 0.5–5 GPa and temperatures between 300 °C and 730 °C). Fluids rich in H_2_ may be common in the upper mantle and generated by fluid-rock or melt-rock reactions^6,6,47^, or dissolved in minerals^34^. Moreover, immiscibility of H_2_ or H_2_-CH_4_ in aqueous fluids in the graphite or diamond stability field^22–25^ could extend the effectiveness of our results to local fluid–mineral interactions in oxygen-bearing systems. Fig. 1 summarizes the main results of this work, in which we propose that hydrogen and different reduced carbon species found in the Earth’s mantle could be an important source of abiogenic hydrocarbons. Our results provide a possible explanation for geological findings of the detection of methane and hydrogen in diamonds extracted from the lower mantle^7,8^. Thus, the different species might contribute to the cycling of deep carbon in the Earth’s upper mantle via methane production and act as sources of deep energy for shallower reservoirs^1,6,48^.

## Methods

Ultra-low fluorescent diamond anvils, with culet diameters ranging between 200 and 300 μm, were used. Re-foil or W-foil gaskets were used to contain the samples. No differences were found when using Re or W, or Au-lined Re gaskets. Research-grade (99.9999%) hydrogen and deuterium samples were gas loaded into DACs at a pressure of 0.2 GPa. Prior to gas loading, the diamond surfaces were thoroughly cleaned with several washes, first with acetone and then with of doubly distilled de-ionized water and the use of organic solvents was avoided. The gasket was first cleaned in water in an ultrasonic bath and then placed on the diamond surface for immediate loading. After loading, samples were mapped with Raman spectroscopy to rule out any possible contamination. High-purity graphite (99.8%, 43078 Alfa Aesar) and glass-like spherical powder by Alpha Aesar were loaded into the DAC, and research-grade hydrogen (99.9999%) was subsequently gas loaded at a pressure of 0.2 GPa. Al_2_O_3_ coatings were done via chemical vapour deposition. High-quality Raman spectra were acquired using a custom-built micro-focused Raman system, using a 514 nm laser as the excitation line. High-temperature experiments were conducted using modified high-temperature Mao–Bell DACs equipped with a primary and a secondary heater, and thermocouples. A type-K thermocouple was partially clamped between the gasket and the diamond anvil. Good mechanical contact ensures a more accurate temperature measurement, while ensuring optimum proximity to the sample chamber. Heating was done in two stages: (i) the primary, external to the cell assembly heating to 500 °C; and (ii) a secondary internal heater, situated around the diamond anvils, heating to 730 °C. The secondary heater made of a Mo-coil heating element was driven by a DC power supply on a feedback loop with a high sampling rate controller^49–51^.

## Supplementary information

Supplementary information

## Data Availability

The data that support the findings of this study are available from the corresponding author upon request.
