# Supplementary material for: In-situ abiogenic methane synthesis from diamond and graphite under geologically relevant conditions
Source: Nat Commun. 2021 Nov 4;12:6387. doi: 10.1038/s41467-021-26664-3 (PMC8569197; doi:10.1038/s41467-021-26664-3)
Supplement: Supplementary file 1 — Supplementary information [file 41467_2021_26664_MOESM1_ESM.pdf]

Supplementary materials for: "In-situ **abiogenic methane synthesis from diamond and graphite under geologically relevant conditions"**

Miriam Peña-Alvarez [1], Alberto Vitale Brovarone[2,3], Mary-Ellen Donnelly[4], Mengnan Wang[1], Philip Dalladay-Simpson[4], Ross Howie[4], Eugene Gregoryanz[1,4,5]

[1]Centre for Science at Extreme Conditions and School of Physics and Astronomy,

University of Edinburgh, Edinburgh, U.K.

[2] Dipartimento di Scienze Biologiche, Geologiche e Ambientali (BiGeA), Alma Mater Studiorum

Università di Bologna, Piazza di Porta San Donato 1, 40126 Bologna, Italy

[3]Sorbonne Université, Muséum National d'Histoire Naturelle, UMR CNRS 7590, IRD, Institut de

Minéralogie, de Physique des Matériaux et de Cosmochimie, IMPMC, 75005 Paris, France

[4]Center for High Pressure Science and Technology Advanced Research, Shanghai, China

[5]Key Laboratory of Materials Physics, Institute of Solid State Physics,

Chinese Academy of Sciences, Hefei, China.

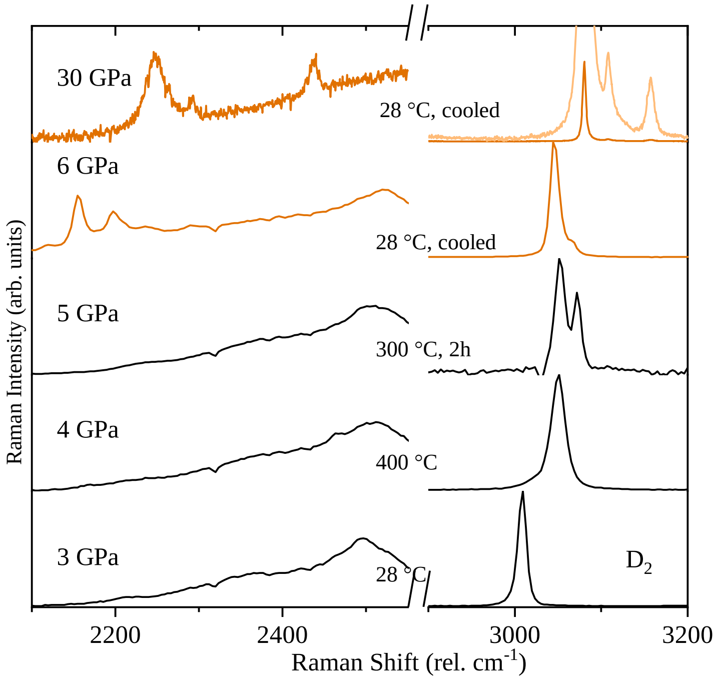

Supplementary Figure 1. Resistive heating of deuterium in diamond anvil cell. During the heating cycle pressure was increasing from 3 to 6 GPa. After quenching pressure was increased to 30 GPa at room temperature to compare the frequencies of the synthesised product with those of deuterated methane (see Supplementary Figure S2).

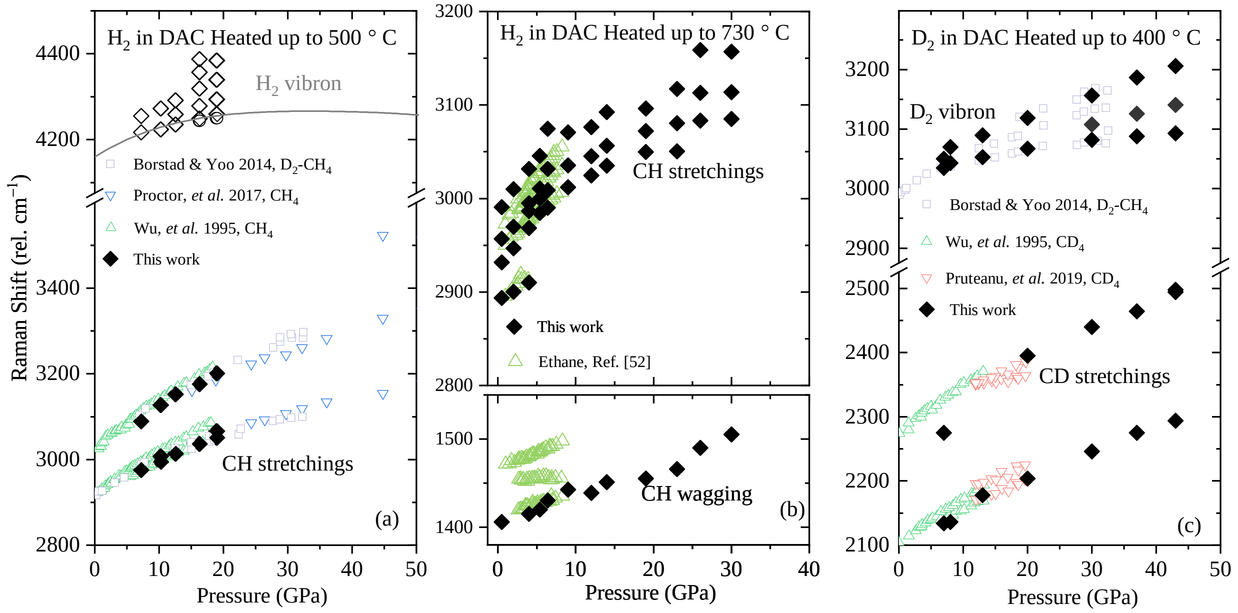

Supplementary Figure 2. Comparison of the Raman frequencies of the synthesised products in our experiments during compression with data from literature. (a) Methane and hydrogen. (b) Ethane. (c)

Deuterated methane. The literature data for comparison are taken from: Wu, *et al* [1] and Pruteanu, *et al.* [2] for CD_4; Borstad, *et al.* [3] and Proctor *et al.* [4] for D_2 and CH_4; Howie, *et al.* [5] for H_2.

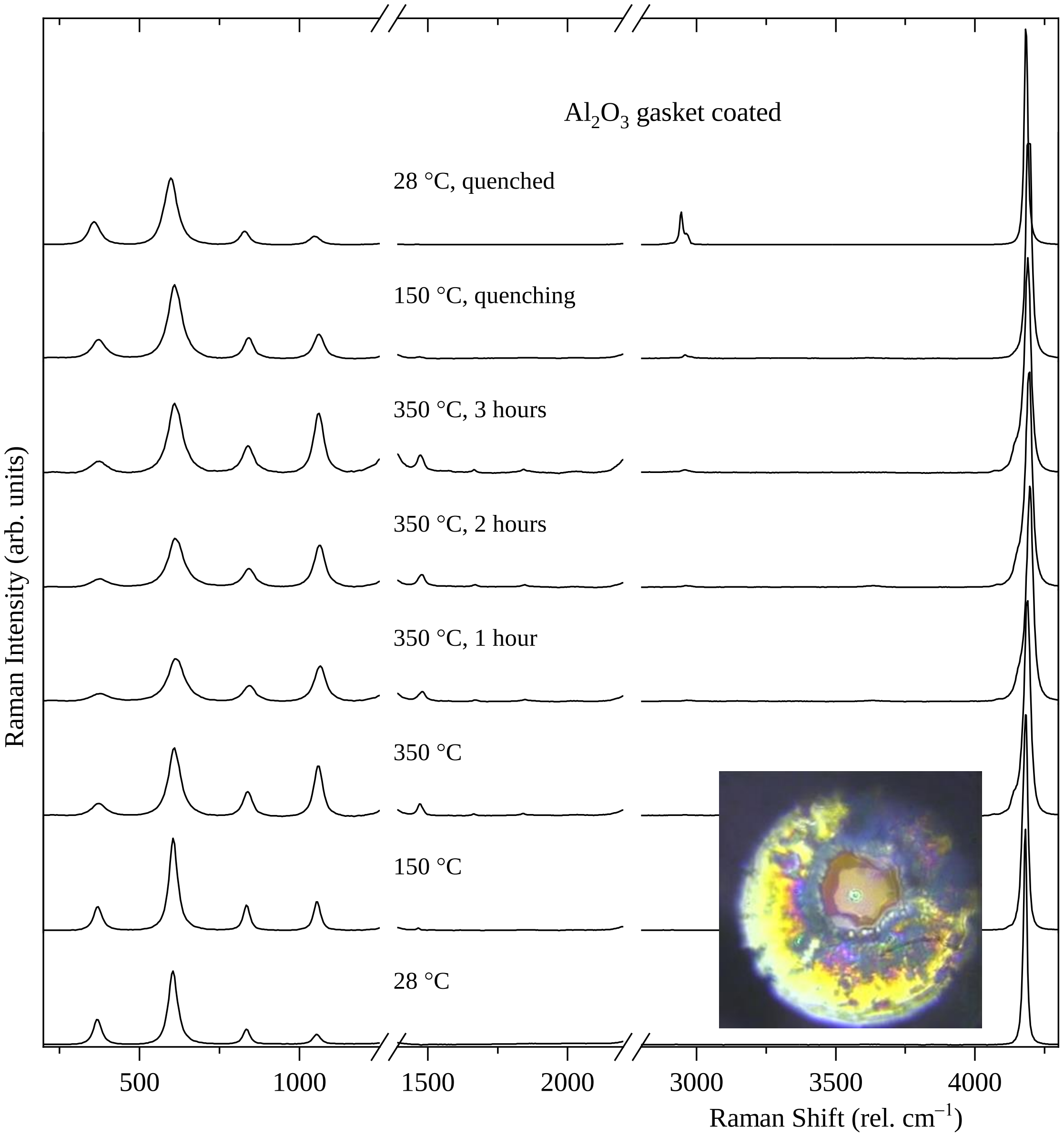

Supplementary Figure 3. Raman spectra of H_2 during a heating cycle at 3 GPa using a coated gasket with an insulating Al_2O_3 layer. In this experiment the rhenium gasket was coated with Al_2O_3 chemically vapour deposited 500 nm thick on both sides.

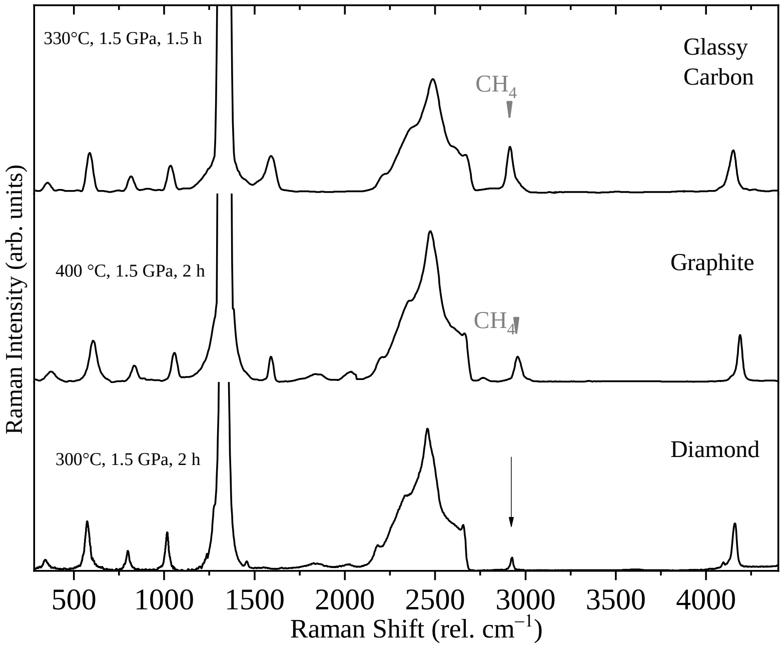

Supplementary Figure 4. Comparison of resistive heating of hydrogen in DAC. From bottom to top: H_2 in the chamber, hydrogen with graphite and glassy carbon with hydrogen.

Supplementary Table 1. Summary of the experimental runs

**N:**	**gas ket**	**Lining to protect gasket**	**max T °C**	**Time of heating run, approx.**	**Pressure during experiment**	**Product**
H_2	Re	Au	705 °C	Kept for 10 min 705 °C	2-4 GPa	CH_4
H_2	Re	Au	605 °C	Kept for 1 hour 605 °C	2-4 GPa	CH_4
H_2	W	Au	375 °C	Kept for 20 min 375 °C	2-4 GPa	CH_4
H_2	Re	Au	550 °C	Kept for 2 hours at 550 °C	1-3 GPa	CH_4
H_2	W	None	600 °C	Kept for 10 min 600 °C	1-3 GPa	CH_4
H_2	Re	None	730 °C	Kept for 2 hours at 500 °C	1-3 GPa	C_2H_8 (ethane)$^-$
H_2	W	None	550 °C	Kept for 2 hours at 400 °C	1-3 GPa	CH_4
H_2	W	None	550 °C	Kept for 2 hours at 400 °C	1-3 GPa	CH_4
H_2	Re	None	550 °C	Kept for 2 hours at 400 °C	0.5-1.8 GPa	CH_4
H_2	Re	None	400 °C	Kept for 3 hours at 400 °C	1-3 GPa	CH_4
H_2	Re	None	400 °C	Kept for 3 hours at 400 °C	1-3 GPa	CH_4
H_2	Re	None	330 °C	Kept for 3 hours at 330 °C	1-2 GPa	CH_4
H_2	Re	Diamonds coated with Al_2O_3 and gasket coated with Al_2O_3	500 °C	Kept for 3 hours at 360 °C	3-4 GPa	Traces of methane after the coating peeled off
H_2	Re	Gasket coated with Al_2O_3	350 °C	Kept for 3 hours at 350 °C	1-4 GPa	CH_4

H_2	Re	Diamonds coated with Al_2O_3	500 °C	Kept for 3 hours at 350 °C	2-4 GPa	Traces of methane after the coating peeled off
[redacted]						
D_2	Re	None	750 °C	Kept for 1 hour at 325 C. T was increased to 750 °C	2-4 GPa	CD_4
D_2	Re	Au	500 °C	Kept for 1 hour at 300 C	2-4 GPa	CD_4
D_2	Re	Au	480 °C	Kept for 2 hours at 480 C	2-4 GPa	CD_4
D_2	W	none	750 °C	Kept for 1 hour at 300 C	2-4 GPa	CD_4

[1] Wu, Y., Sasaki, S. & Shimizu, H. High-pressure Raman study of dense methane: CH4 and CD4. Journal of Raman Spectroscopy 26, 963–967 (1995).
[2] Pruteanu, C. G. The effect of deuteration on the optical spectra of compressed methane. AIP Advances 9, 045033 (2019).
[3] Borstad, G. M. & Yoo, C.-S. Hydrogen bonding induced proton exchange reactions in dense D2-NH3 and D2-CH4 mixtures. The Journal of Chemical Physics 140, 044510 (2014).
[4] Proctor, J. E., Maynard Casely, H. E., Hakeem, M. A. & Cantiah, D. Raman spectroscopy of methane (CH4) to 165 GPa: Effect of structural changes on Raman spectra. Journal of Raman Spectroscopy 48, 1777 – 1782 (2017).
[5] Howie, R. T., Guillaume, C. L., Scheler, T., Goncharov, A. F., Gregoryanz, E. Mixed molecular and atomic phase of dense hydrogen. Physical Review Letters 108, 125501 (2012).
